# Finerenone exposure and ischaemic stroke in patients with type 2 diabetes and chronic kidney disease: A propensity score-matched cohort study

**DOI:** 10.1016/j.clinme.2026.100617

**Published:** 2026-08-06

**Authors:** Wu-Lung Chuang, Ruey-Hwang Chou, Yuan-Hung Wang, I-Ju Tsai, Cheng-Li Lin, Der-Yang Cho, Kuang-Hsi Chang

**Affiliations:** a Division of Endocrinology and Metabolism, Department of Internal Medicine, Changhua Christian Hospital, Changhua 500, Taiwan; b Division of Endocrinology and Metabolism, Department of Internal Medicine, Lukang Christian Hospital, Changhua 505, Taiwan; c Graduate Institute of Biomedical Sciences, China Medical University, Taichung 404, Taiwan; d Center for Molecular Medicine, China Medical University Hospital, Taichung 404, Taiwan; e Department of Medical Laboratory and Biotechnology, Asia University, Taichung 413, Taiwan; f Graduate Institute of Clinical Medicine, College of Medicine, Taipei Medical University, Taipei 110, Taiwan; g Department of Medical Research, Shuang Ho Hospital, Taipei Medical University, New Taipei City 235, Taiwan; h Management Office for Health Data, China Medical University Hospital, Taichung 404, Taiwan; i College of Medicine, China Medical University, Taichung 404, Taiwan; j Translational Cell Therapy Center, Department of Medical Research, China Medical University Hospital, Taichung 40447, Taiwan; k Department of Neurosurgery, China Medical University Hospital, Taichung 40447, Taiwan; l Graduate Institute of Biomedical Sciences, China Medical University, Taichung 40447, Taiwan; m Department of Long-Term Care, College of Nursing, Asia University, Taichung 413, Taiwan

**Keywords:** Finerenone, Ischaemic stroke, Type 2 diabetes mellitus, Chronic kidney disease, Real-world evidence

## Abstract

**Background:**

Type 2 diabetes mellitus (T2DM) and chronic kidney disease (CKD) commonly coexist and are associated with an increased risk of ischaemic stroke. Finerenone has demonstrated benefits on composite renal and cardiovascular outcomes in randomised trials; however, ischaemic stroke has generally been evaluated as part of composite endpoints, and real-world evidence focusing on this outcome remains limited.

**Methods:**

We conducted a retrospective cohort study using the TriNetX Global Research Network to evaluate the association between finerenone use and incident ischaemic stroke among adults with newly diagnosed T2DM who subsequently developed CKD. Finerenone users were compared with non-users. Propensity score matching was applied to balance demographic characteristics and baseline comorbidities. Cox proportional hazards models were used to estimate hazard ratios (HRs) with 95% confidence intervals (CIs).

**Results:**

A total of 125,086 eligible patients were identified before matching, including 1,990 finerenone users. After 1:1 propensity score matching, 1,990 finerenone users were matched with 1,990 non-users with adequate covariate balance. Finerenone use was associated with a lower observed hazard of ischaemic stroke both before matching (adjusted HR, 0.30; 95% CI, 0.24–0.36) and after matching (adjusted HR, 0.33; 95% CI, 0.26–0.42).

**Conclusions:**

In this real-world cohort of patients with T2DM and CKD, finerenone use was associated with a lower observed hazard of ischaemic stroke.

## Highlights

• Real-world TriNetX cohort assessed finerenone and incident ischaemic stroke in T2DM with CKD.
• Propensity score matched achieved good covariate balance between finerenone users and non-users.
• Finerenone use was associated with lower observed ischaemic stroke risk before matching (adjusted hazard ratio: 0.30).
• The inverse association persisted after matching (adjusted hazard ratio: 0.33), with consistent Kaplan–Meier separation.
• Findings provide hypothesis-generating cerebrovascular evidence beyond composite trial endpoints.

## Introduction

Type 2 diabetes mellitus (T2DM) and chronic kidney disease (CKD) frequently coexist and together contribute to a heightened risk of adverse cardiovascular and cerebrovascular outcomes.^1^, ^2^ Patients affected by both conditions often present with multiple vascular risk factors, and ischaemic stroke remains a significant cause of morbidity, functional impairment and healthcare utilisation in this population.^3^, ^4^, ^5^, ^6^ Although strategies targeting glycaemic control and renal protection have evolved, residual cerebrovascular risk persists among patients with T2DM and CKD.^7^, ^8^, ^9^

Recently, the concept of cardio-kidney-metabolic (CKM) syndrome has been proposed to describe the interrelated pathophysiology linking metabolic risk factors, chronic kidney disease and cardiovascular disease.^10^ Within this framework, patients with T2DM and CKD represent a clinically important high-risk phenotype characterised by overlapping mechanisms, including chronic inflammation, endothelial dysfunction, oxidative stress, vascular stiffness and fibrosis.^10^ These interconnected abnormalities may contribute not only to progressive cardiorenal disease, but also to an increased risk of cerebrovascular events, including ischaemic stroke.^6^, ^11^, ^12^ Framing the present population within the CKM paradigm therefore provides a more clinically meaningful context for understanding stroke risk in patients with T2DM and CKD.

Finerenone, a non-steroidal mineralocorticoid receptor antagonist, may be particularly relevant in the setting of CKM syndrome because mineralocorticoid receptor overactivation has been implicated in inflammation, fibrosis, endothelial dysfunction and cardiorenal injury.^13^, ^14^, ^15^, ^16^ While these trials provide essential evidence regarding overall cardiovascular outcomes, ischaemic stroke has generally been evaluated as part of broader composite endpoints rather than as an isolated outcome.^13^, ^17^, ^18^ Consequently, the relationship between finerenone use and ischaemic stroke has not been fully characterised, particularly in routine clinical settings.^19^, ^20^

Given the clinical importance of ischaemic stroke in patients with T2DM and CKD and the limited real-world evidence addressing this outcome, further investigation is warranted. Using data from the TriNetX Global Research Network, we conducted a retrospective cohort study to evaluate the association between finerenone exposure and subsequent ischaemic stroke among patients with newly diagnosed T2DM who developed CKD. Propensity score (PS) matching was applied to account for measured baseline differences between treatment groups and to reduce potential confounding.

## Methods

### Data source

This retrospective cohort study was conducted using the TriNetX Global Research Network, a federated research platform that provides access to de-identified electronic health record data from participating healthcare organisations. Data in this study were obtained from the TriNetX Global Collaborative Network, with data extracted on 10 December 2025, which defines the end of the follow-up period. Available data include patient demographics, diagnoses coded using the International Classification of Diseases, 10th Revision (ICD-10), and medication records. All analyses were performed within the TriNetX environment in accordance with its data governance framework.

### Study design and study population

A cohort design was applied to identify adult patients with new-onset T2DM (ICD10-CM-code: E08, E09, E11, E13) who subsequently received a diagnosis of CKD (ICD10-CM-code: N18). To restrict the cohort to newly diagnosed T2DM, individuals with any prior T2DM diagnosis before July 2021 were excluded. Eligible patients were identified from July 2021 onward. The index date was defined as the date of the first recorded diagnosis of T2DM. Patients were required to have a documented diagnosis of CKD after the index date.

**Fig. 1:**
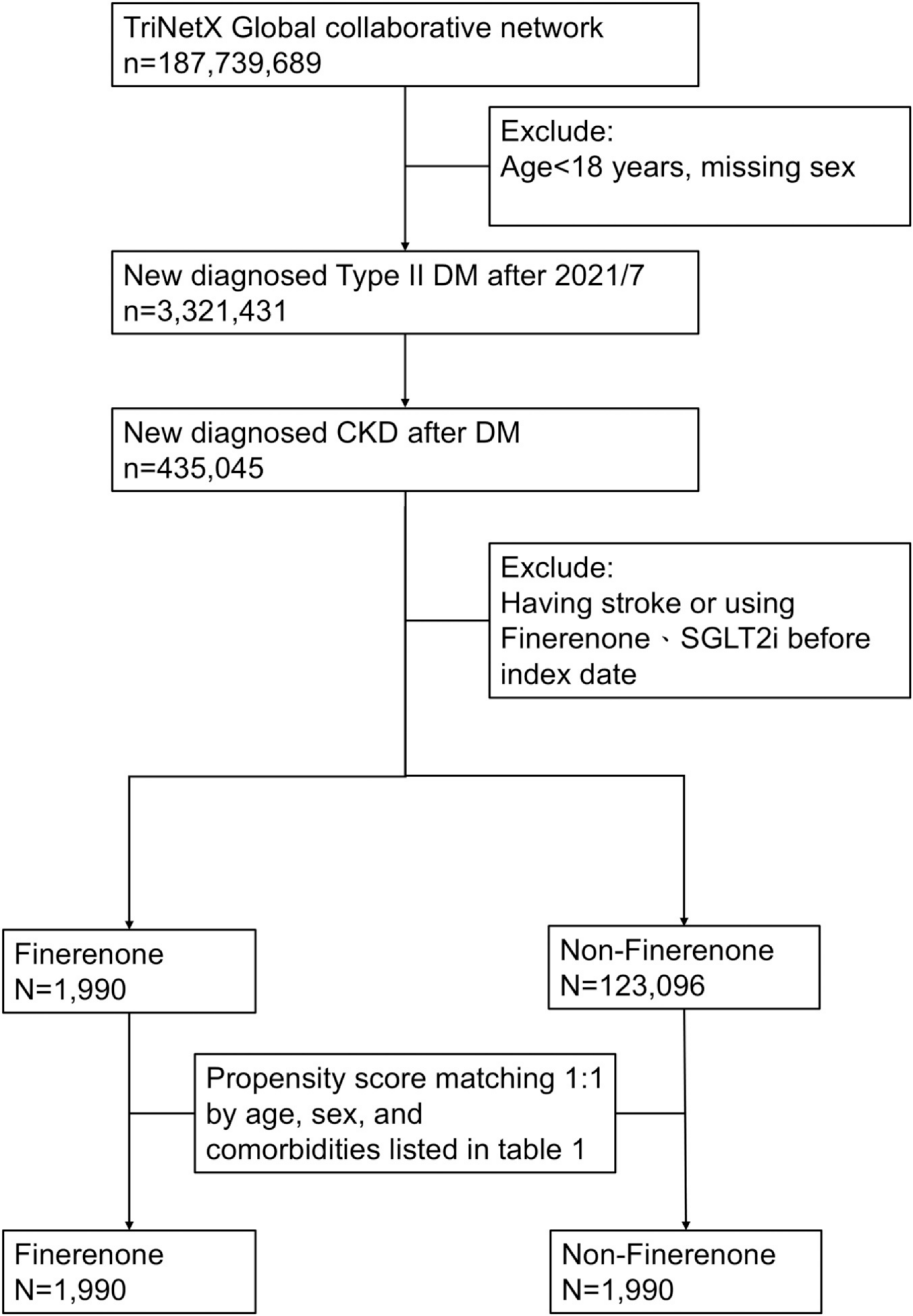
Study flow diagram of cohort selection.

Fig. 1 illustrates the patient selection process and 1:1 PS matching of finerenone and non-finerenone cohorts in the ischaemic stroke study.

### Exposure classification

Patients were classified according to finerenone exposure after the index date. The finerenone cohort comprised individuals with at least one recorded prescription for finerenone after the index date. In contrast, the non-finerenone cohort included patients with no record of finerenone use during the entire observation period. To enhance cohort comparability and ensure a precise temporal sequence, several exclusion criteria were applied. Patients were excluded if they were younger than 18 years at the index date, had missing sex information, or had received finerenone or sodium-glucose cotransporter-2 (SGLT2) inhibitors before the index date. In addition, individuals with a diagnosis of CKD preceding the diagnosis of T2DM were excluded to maintain temporal clarity of disease onset. Finally, patients with a history of ischaemic stroke or transient ischaemic attack before the index date were excluded, as identified by ICD-10 codes I60-I69, G45 and G46, to minimise potential confounding from pre-existing cerebrovascular disease.

### Definitions of outcome and comorbidities

The primary outcome was incident ischaemic stroke, identified using ICD-10 code I63, occurring after the index date. Baseline characteristics assessed at or before the index date included age, sex and comorbid conditions: hypertension (HT, I10-I15), dyslipidaemia (disorders of lipoprotein metabolism and other lipidaemias, E78), obesity (E66), malignancy (C00-D49), heart failure (I50), atrial fibrillation or flutter (I48), asthma (J45), chronic obstructive pulmonary disease (COPD, J44), acute myocardial infarction (AMI, I21), and chronic liver disease (alcoholic liver disease, K70; chronic hepatitis, not elsewhere classified, K73; fibrosis and cirrhosis of liver, K74).

### Statistical analysis

Baseline characteristics were summarised and compared between cohorts using standardised mean differences (SMDs), with an SMD <0.1 indicating acceptable covariate balance. To mitigate measured confounding, 1:1 PS matching was conducted using age, sex and baseline comorbidities. The association between finerenone exposure and ischaemic stroke was evaluated using Cox proportional hazards regression, and results were expressed as hazard ratios (HRs) with 95% confidence intervals (CIs). The proportional hazards assumption was formally tested and satisfied (p = 0.5278). Analyses were performed both before and after PS matching. Statistical significance was assessed using two-sided p-values, with p < 0.05 considered statistically significant.

Due to the platform structure and the use of PS matching to balance baseline characteristics, additional covariate adjustment in the Cox models was not performed; this limitation is inherent to the TriNetX analysis platform. Missing covariate data were automatically excluded by the platform, and no imputation was performed.

## Results

A total of 125,086 patients with T2DM and CKD were identified before PS matching, including 1,990 finerenone users and 123,096 non-finerenone users. Before matching, finerenone users were younger (mean age 65.2 vs. 68.4 years; SMD = 0.29) and had a lower prevalence of several cardiovascular comorbidities, including heart failure and atrial fibrillation, indicating baseline imbalance between the two groups. After 1:1 PS matching, 1,990 finerenone users were successfully matched with 1,990 non-finerenone users. All baseline demographic characteristics and comorbidities were well balanced after matching, with standardised mean differences below 0.1 for all variables, confirming adequate covariate balance (Table 1).

**Table 1:** Baseline patient characteristics.

Characteristic	Before PS matching			After PS matching		
	Finerenone	Non-finerenone	SMD	Finerenone	Non-finerenone	SMD
	n = 1,990	n = 123,096		n = 1,990	n = 1,990	
Age at index, years						
Mean (SD)	65.2 (12.0)	68.4 (11.6)	0.29	65.2 (12.0)	65.3 (11.8)	0.01
Sex, n (%)						
Male	1,189 (59.7)	71,276 (57.9)	0.03	1,189 (59.7)	1,182 (59.4)	0.01
Female	801 (40.3)	51,820 (42.1)		801 (40.3)	808 (40.6)	
Comorbidities, n (%)						
Hypertensive diseases (I10-I15)	329 (16.5)	23,304 (18.9)	0.09	329 (16.5)	325 (16.3)	0.01
Dyslipidaemia (E78)	201 (10.1)	15,814 (12.8)	0.11	201 (10.1)	200 (10.1)	0.00
Neoplasms (C00-D49)	198 (10.0)	12,870 (10.5)	0.01	198 (10.0)	206 (10.4)	0.01
Overweight and obesity (E66)	105 (5.3)	8,164 (6.6)	0.08	105 (5.3)	108 (5.4)	0.01
Heart failure (I50)	51 (2.6)	7,394 (6.0)	0.20	51 (2.6)	50 (2.5)	0.00
Atrial fibrillation and flutter (I48)	37 (1.9)	5,774 (4.7)	0.18	37 (1.9)	38 (1.9)	0.00
Asthma (J45)	33 (1.7)	3,299 (2.7)	0.07	33 (1.7)	31 (1.6)	0.01
COPD (J44)	30 (1.5)	4,094 (3.3)	0.13	30 (1.5)	30 (1.5)	0.00
AMI (I21)	15 (0.8)	2,446 (2.0)	0.06	15 (0.8)	13 (0.7)	0.01
Alcoholic liver disease (K70)	10 (0.5)	453 (0.4)	0.11	10 (0.5)	10 (0.5)	0.00
Chronic hepatitis (K73)	10 (0.5)	119 (0.1)	0.04	10 (0.5)	10 (0.5)	0.00
Fibrosis and cirrhosis of liver (K74)	10 (0.5)	1,195 (1.0)	0.05	10 (0.5)	10 (0.5)	0.00

PS, propensity score; SMD, Standardised Mean Difference; COPD, Other chronic obstructive pulmonary disease; AMI, Acute myocardial infarction.

The outcome analyses were restricted to incident events. For each outcome of interest, patients with a documented history of the corresponding condition before the index date were excluded to ensure that only newly occurring events were evaluated. Consequently, the number of patients at risk and thus the final analytic sample size varied slightly across outcome analyses. It may differ from the population presented in Table 1, depending on the prevalence of pre-existing conditions in the study cohort. During follow-up, finerenone use was associated with a significantly lower risk of ischaemic stroke. In the unmatched cohort, ischaemic stroke occurred in 105 of 2,162 finerenone users and 16,529 of 116,549 non-finerenone users, corresponding to an adjusted hazard ratio (HR) of 0.30 (95% CI, 0.24–0.36). After PS matching, 98 ischaemic stroke events were observed among 1,943 finerenone users, compared with 238 events among 1,791 matched non-finerenone users. In the matched analysis, finerenone use remained associated with a substantially lower risk of ischaemic stroke (adjusted HR, 0.33; 95% CI, 0.26–0.42) (Table 2). These findings indicate a consistent and robust inverse association between finerenone use and ischaemic stroke risk across both unmatched and matched analyses.

**Table 2:** Association between finerenone and ischaemic stroke.

Model	Finerenone		Non-Finerenone		Adj. HR (95% CI)
	N	Event	N	Event	
After PS matching	1,943	98	1,791	238	0.33 (0.26,0.42)
Before PS matching	2,162	105	116,549	16,529	0.30 (0.24,0.36)

Event, number of patients with ischaemic stroke; Adj. HR, adjusted hazard ratio, adjusted for age, gender, and comorbidities; CI, confidence interval.

**Fig. 2:**
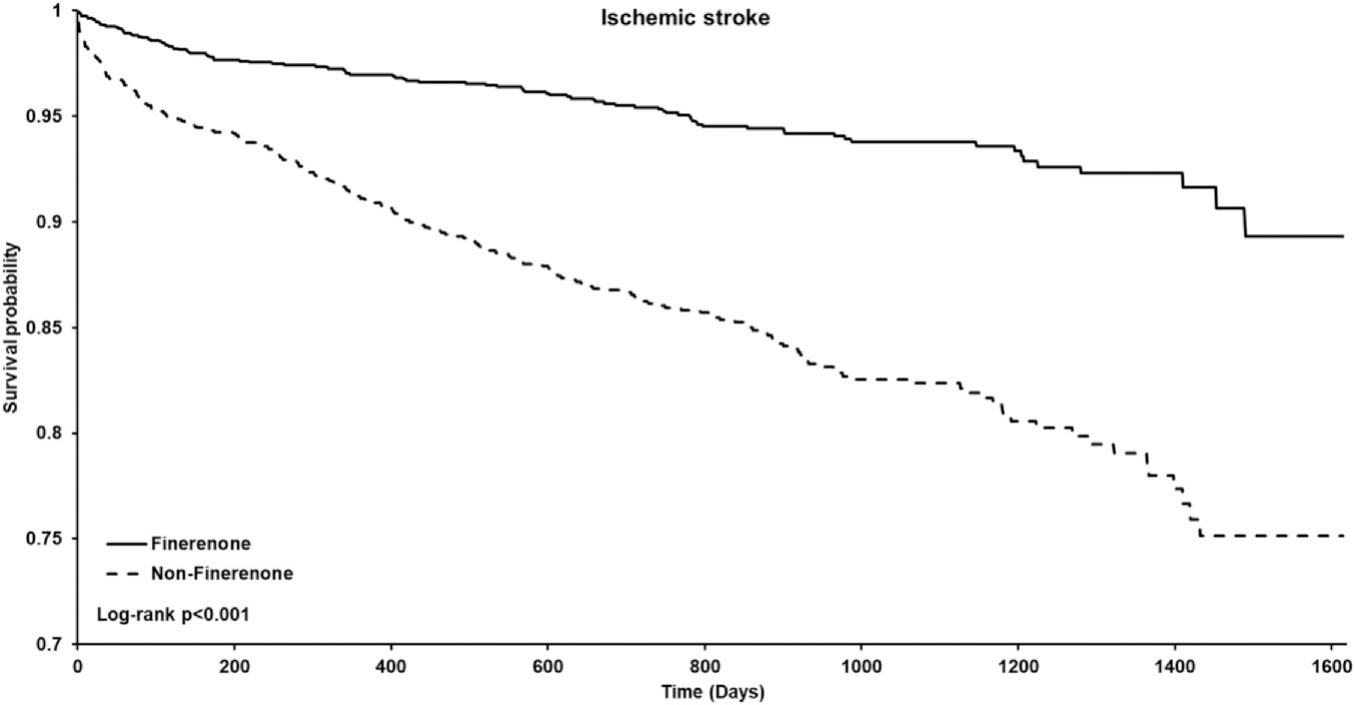
Kaplan–Meier survival curves for ischaemic stroke-free probability in finerenone users versus nonusers.

Kaplan–Meier curves showed an early and sustained divergence between the finerenone and non-finerenone cohorts, with finerenone users exhibiting a consistently higher probability of remaining free of ischaemic stroke during follow-up. The difference between curves was statistically significant on log-rank testing (p < 0.001), in agreement with the findings from the Cox proportional hazards models (Fig. 2).

## Discussion

In this large real-world cohort of patients with newly diagnosed T2DM who subsequently developed CKD, finerenone use was associated with a lower observed hazard of ischaemic stroke compared with non-use. This association was consistently observed before and after PS matching and persisted after adjustment for demographic characteristics and baseline comorbidities. Large real-world healthcare databases provide an opportunity to examine associations between medication exposure and clinical outcomes that may be infrequent or not explicitly evaluated as standalone endpoints in randomised clinical trials. By capturing heterogeneous patient populations, more extended observation periods and routine prescribing patterns, such data can complement evidence derived from controlled trials. However, because real-world analyses are observational in nature and susceptible to residual confounding, the present findings should be interpreted as associative rather than indicative of a causal relationship.

Randomised clinical trials of finerenone have demonstrated favourable effects on composite renal and cardiovascular outcomes in patients with T2DM and CKD.^13^, ^17^, ^18^ However, individual cerebrovascular events, including ischaemic stroke, have generally been evaluated as components of broader composite cardiovascular endpoints rather than as standalone outcomes.^19^, ^20^ As a result, the specific relationship between finerenone exposure and ischaemic stroke risk has not been fully characterised. By focusing explicitly on ischaemic stroke in a real-world setting, the present study extends the evidence from trials and provides clinically relevant information reflective of routine practice.

Finerenone is a non-steroidal mineralocorticoid receptor antagonist with anti-inflammatory and antifibrotic properties that have been implicated in ameliorating mineralocorticoid receptor-mediated vascular inflammation and fibrosis, which are key contributors to vascular dysfunction and atherosclerosis in diabetes and chronic kidney disease.^21^, ^22^ Overactivation of the mineralocorticoid receptor has been linked to oxidative stress and endothelial dysfunction in preclinical models, suggesting that antagonism of this pathway might favourably influence endothelial health and thereby modulate cerebrovascular risk.^23^, ^24^ In addition, finerenone has been shown in basic and clinical studies to reduce markers of cardiorenal stress and improve outcomes related to cardiovascular structure and function, which may indirectly reflect systemic vascular effects relevant to stroke risk.^25^, ^26^ However, cerebrovascular endpoints, such as ischaemic stroke, were not evaluated as primary outcomes in the major randomised finerenone trials, and it remains uncertain whether these mechanistic pathways translate into a specific reduction in ischaemic stroke incidence.^24^ Therefore, the present findings should be regarded as hypothesis-generating, and prospective studies with detailed mechanistic and cerebrovascular outcome data are needed to clarify the biological pathways through which finerenone may influence cerebrovascular risk.

Several factors may contribute to the observed association. Patients prescribed finerenone may differ systematically from those not receiving the medication with respect to underlying cardiovascular risk profiles, intensity of clinical monitoring, or use of concomitant therapies. Although PS matching was used to balance measured baseline characteristics, residual confounding from unmeasured factors cannot be excluded. Accordingly, the observed association should be interpreted cautiously and not as evidence of a protective or causal effect.

This study has notable strengths, including the use of an extensive, multicentre real-world database and the inclusion of patients managed in routine clinical care.^27^ Nevertheless, several limitations warrant consideration. First, the observational design precludes causal inference.^28^ In addition, the present study did not adopt an active comparator design, and finerenone users were compared with non-users rather than with patients receiving another mineralocorticoid receptor antagonist. This may have increased the possibility of residual confounding, including confounding by indication and treatment-selection bias. Healthy user bias, differences in healthcare engagement and competing risks may also have influenced the magnitude of the observed hazard ratio. Second, exposure and outcome ascertainment relied on ICD-10 diagnostic codes and prescription records, which are primarily collected for administrative and clinical purposes and may be subject to coding variability and misclassification across institutions.^29^ Prescription records also do not capture actual medication intake, adherence or treatment duration.^30^ In addition, socioeconomic factors, particularly in settings where finerenone is not yet available as a generic medication, may have influenced access to or affordability of finerenone and could not be fully addressed through PS matching in the present study. Third, detailed laboratory data, such as kidney function measures, blood pressure, glycaemic control and inflammatory markers, were not consistently available and therefore could not be incorporated into the analysis.^27^ Fourth, the follow-up duration may not have been sufficient to capture all ischaemic stroke events. In some patients with diabetes, CKD progression and the subsequent occurrence of ischaemic stroke may develop over a prolonged period. Accordingly, ischaemic stroke events that occurred beyond the available observation window could not be captured in this study, which may have resulted in an underestimation of late ischaemic stroke events. Finally, although the TriNetX network includes data from multiple healthcare organisations, the generalisability of these findings to other populations or healthcare systems should be interpreted with caution.^31^ In addition, because participating in healthcare organisations may include academic or referral-based centres, and because the number of finerenone users was relatively limited, these patients may have undergone closer monitoring or received more intensive care than the broader population of patients with diabetes and incident CKD.

Moreover, variability in global approval timelines and real-world uptake of finerenone may influence exposure patterns and thereby affect the observed associations, representing an additional potential limitation of this study. Although laboratory measures such as eGFR are available in the TriNetX platform, CKD was defined in this study using ICD codes to ensure consistency, reproducibility and inclusion of a broad real-world patient population. We acknowledge that the absence of laboratory-based staging represents a limitation.

## Conclusion

Despite these limitations, the present study has several features that support the reliability and relevance of its findings. The use of an extensive, multicentre real-world database reduces the likelihood of single-centre selection bias and enhances the representation of patients managed in routine clinical practice. In addition, the application of PS matching and multivariable adjustment helped balance measured baseline characteristics between comparison groups, thereby strengthening the robustness of the observed associations. Moreover, by specifically examining ischaemic stroke, an outcome not routinely evaluated as a standalone endpoint in prior randomised trials of finerenone, this study provides complementary real-world evidence that may inform future prospective investigations. Accordingly, while the findings should be interpreted as associative rather than causal, they offer clinically relevant insights and may serve as a foundation for further hypothesis-driven research.

## CRediT authorship contribution statement

**Kuang-Hsi Chang:** Writing – review & editing, Writing – original draft, Data curation. **Cheng-Li Lin:** Software, Formal analysis. **Der-Yang Cho:** Validation, Resources. **Wu-Lung Chuang:** Writing – original draft, Conceptualization. **Ruey-Hwang Chou:** Validation. **Yuan-Hung Wang:** Validation. **I-Ju Tsai:** Software, Formal analysis.

## Ethics approval and consent to participate

All analyses were conducted using de-identified data. In accordance with TriNetX policies, institutional review board approval and informed consent were not required, as no patient-identifiable information was accessed.

## Funding

This study is supported in part by the Taiwan Ministry of Health and Welfare Clinical Trial Center (MOHW113-TDU-B-212-114009), Lukang Christian Hospital (Y_115_0038), and 10.13039/501100004391China Medical University Hospital (DMR-111-105; DMR-112-087; DMR-113-009; DMR-113-156; DMR-114-046; DMR-114-139; DMR-115-161). We are grateful to the Health Data Science Center at China Medical University Hospital for providing administrative, technical, and financial support.

## Declaration of Competing Interest

The authors declare that they have no known competing financial interests or personal relationships that could have appeared to influence the work reported in this paper.

## Data Availability

The data supporting the findings of this study were obtained from the TriNetX Global Research Network (https://trinetx.com), with data extracted on 10 December 2025, which defined the end of the follow-up period. The data are de-identified and accessible to users with institutional agreements with TriNetX. Due to licensing restrictions, the raw data cannot be shared publicly.
